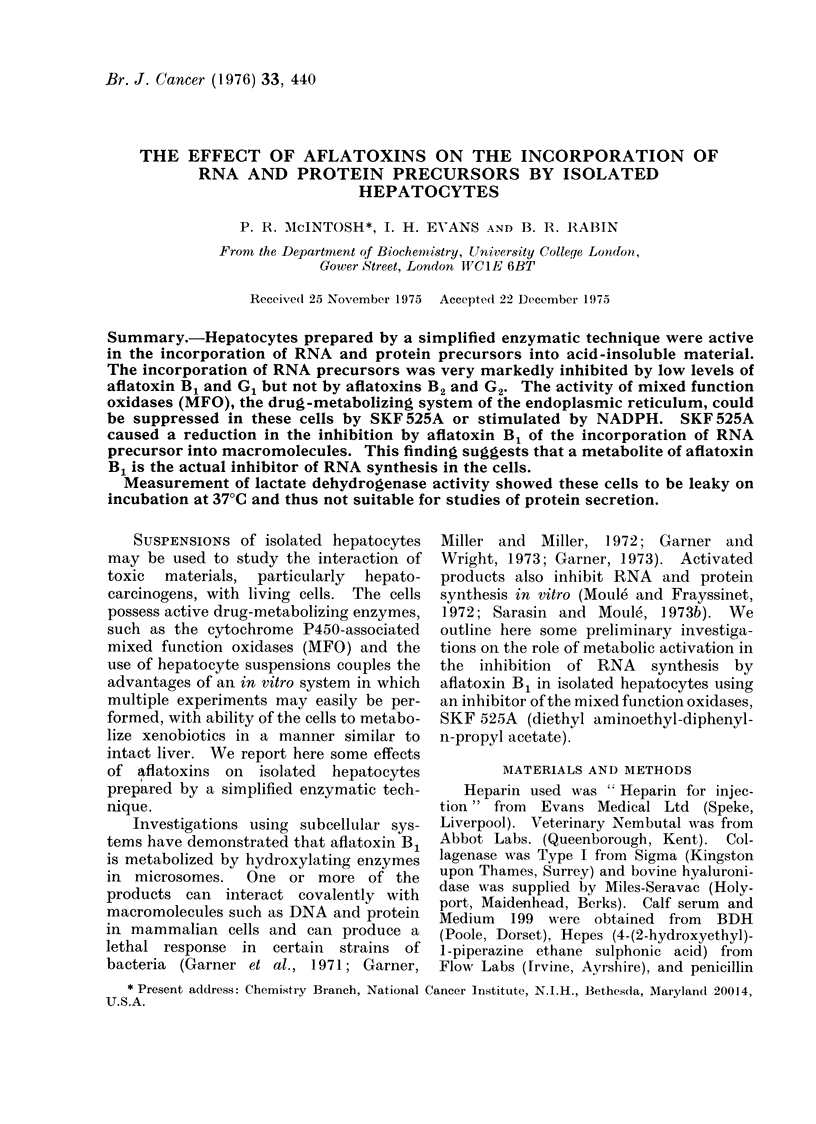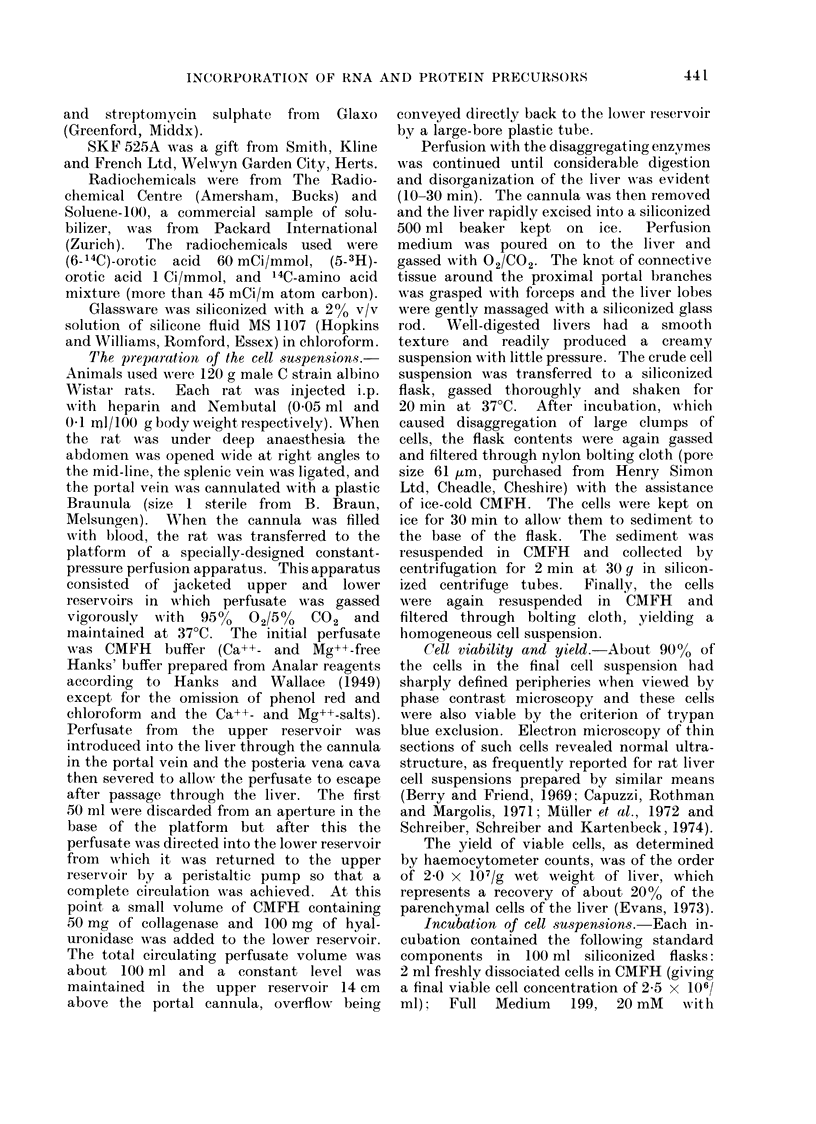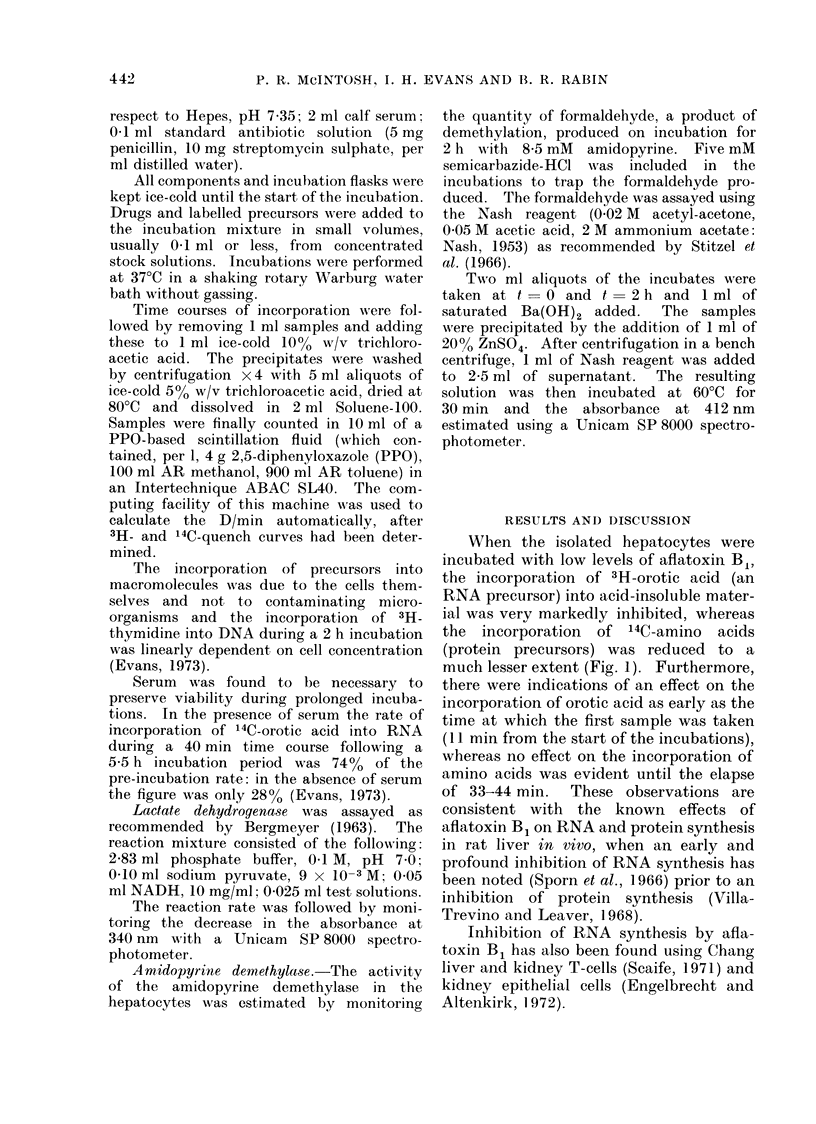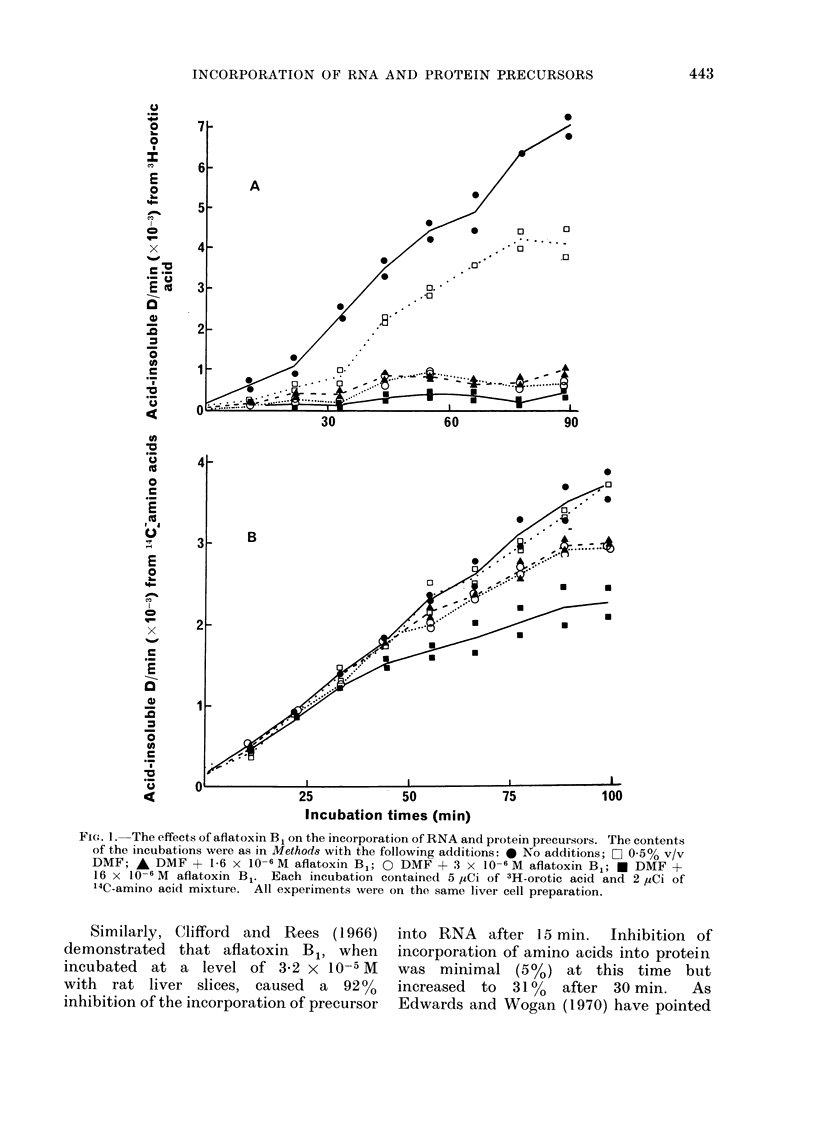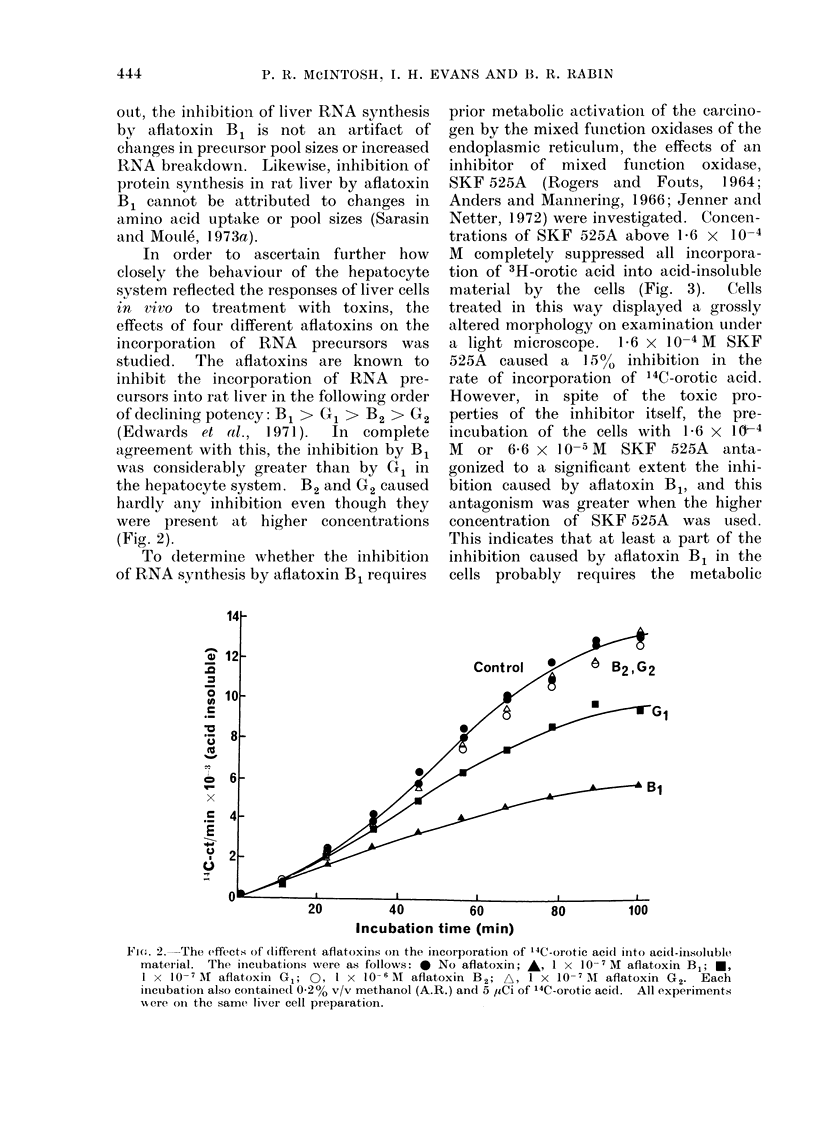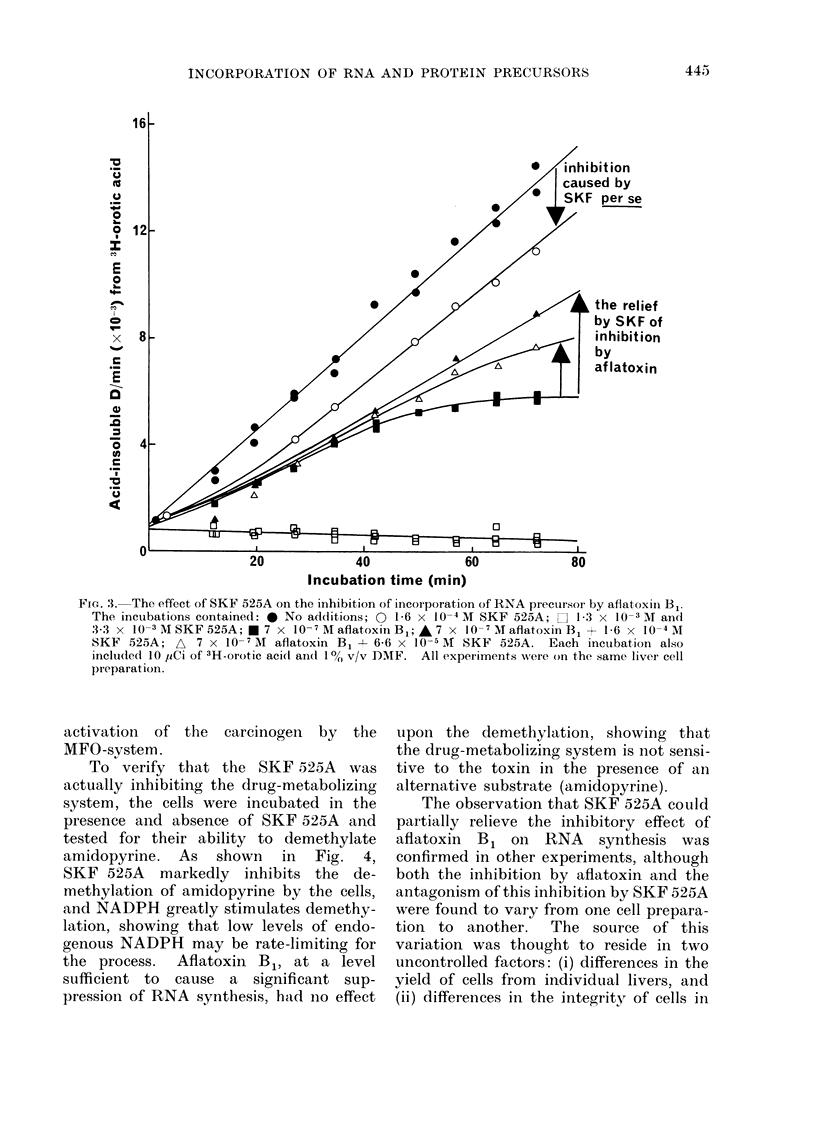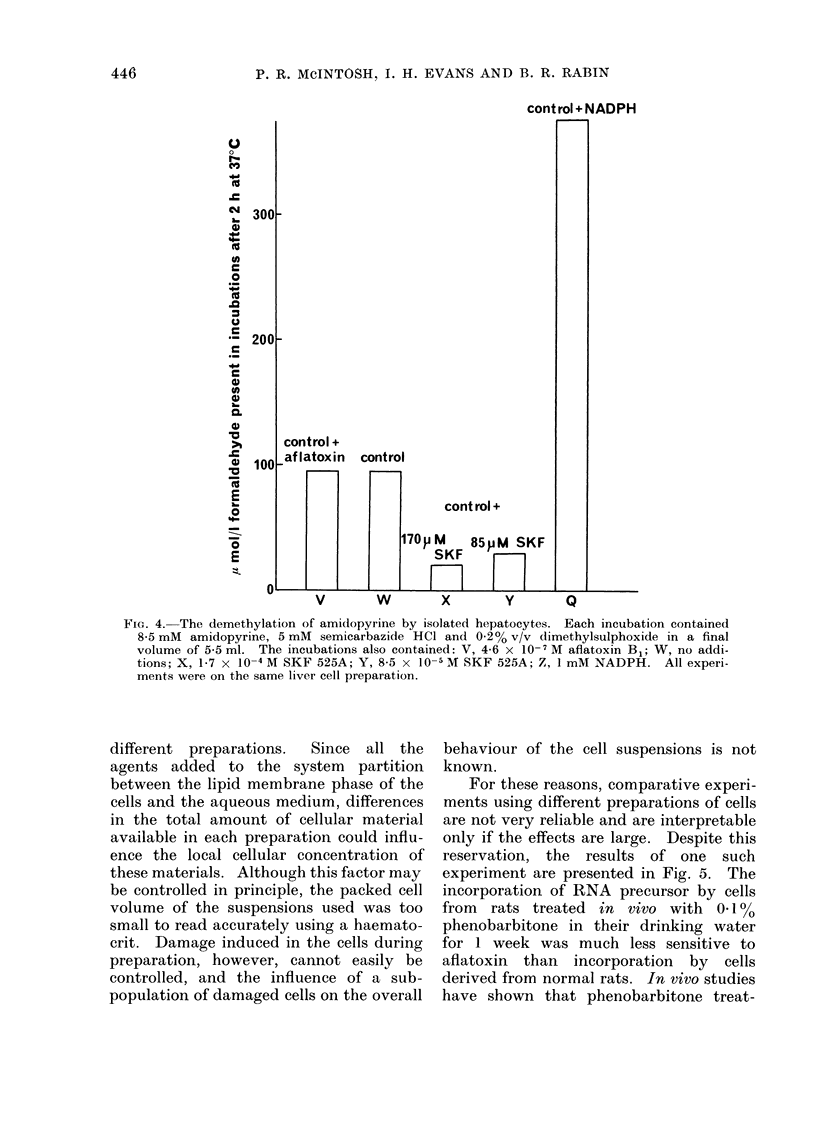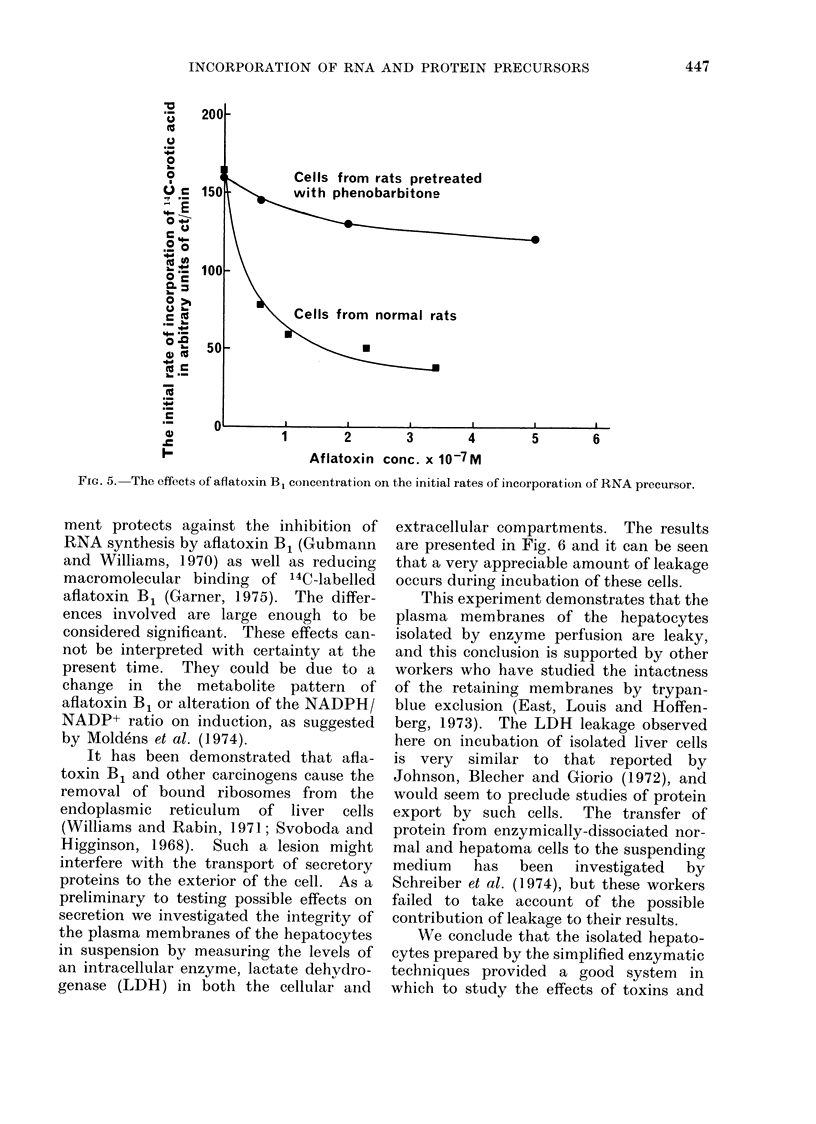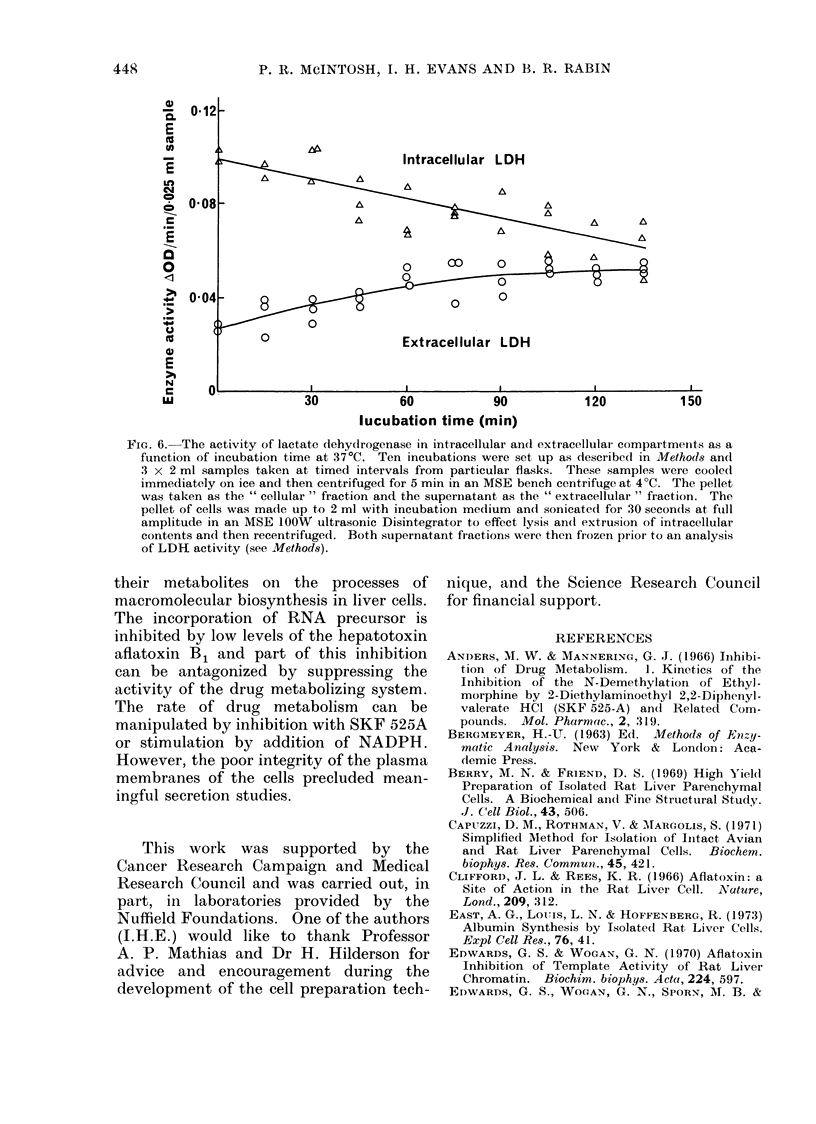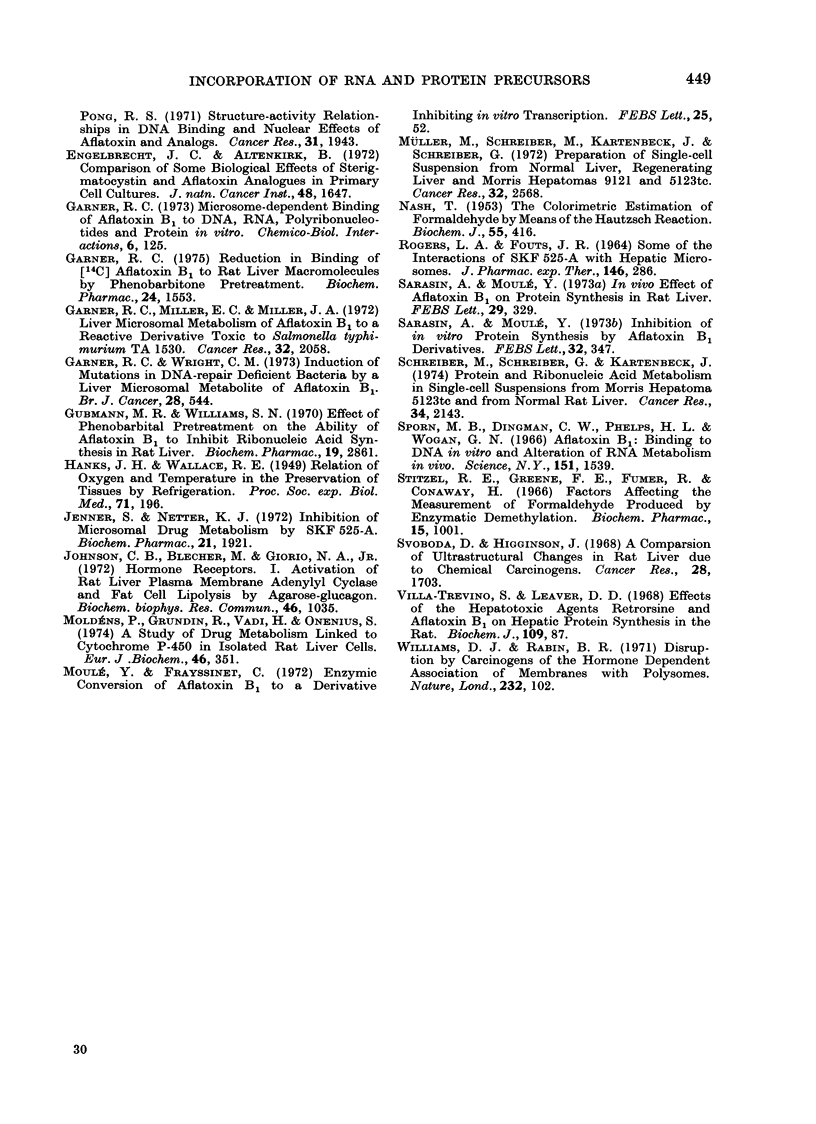# The effect of aflatoxins on the incorporation of RNA and protein precursors by isolated hepatocytes.

**DOI:** 10.1038/bjc.1976.69

**Published:** 1976-04

**Authors:** P. R. McIntosh, I. H. Evans, B. R. Rabin

## Abstract

Hepatocytes prepared by a simplified enzymatic technique were active in the incorporation of RNA and protein precursors into acid-insoluble material. The incorporation of RNA precursors was very markedly inhibited by low levels of aflatoxin B1 and G1 but not by aflatoxins B2 and G2. The activity of mixed function oxidases (MFO), the drug-metabolizing system of the endoplasmic reticulum, could be suppressed in these cells by SKF525A or stimulated by NADPH. SKF525A caused a reduction in the inhibition by aflatoxin B1 of the incorporation of RNA precursor into macromolecules. This finding suggests that a metabolite of aflatoxin B1 is the actual inhibitor of RNA synthesis in the cells. Measurement of lactate dehydrogenase activity showed these cells to be leaky on incubation at 37 degrees C and thus not suitable for studies of protein secretion.


*Br. J. Cancer* (1976) **33**, 440

### THE EFFECT OF AFLATOXINS ON THE INCORPORATION OF RNA AND PROTEIN PRECURSORS BY ISOLATED HEPATOCYTES

P. R. McINTOSH*, I. H. EVANS AND B. R. RABIN

*From the Department of Biochemistry, University College London, Gower Street, London WC1E 6BT*

Received 25 November 1975 Accepted 22 December 1975

**Summary.—Hepatocytes prepared by a simplified enzymatic technique were active in the incorporation of RNA and protein precursors into acid-insoluble material. The incorporation of RNA precursors was very markedly inhibited by low levels of aflatoxin $B_1$ and $G_1$ but not by aflatoxins $B_2$ and $G_2$. The activity of mixed function oxidases (MFO), the drug-metabolizing system of the endoplasmic reticulum, could be suppressed in these cells by SKF 525A or stimulated by NADPH. SKF 525A caused a reduction in the inhibition by aflatoxin $B_1$ of the incorporation of RNA precursor into macromolecules. This finding suggests that a metabolite of aflatoxin $B_1$ is the actual inhibitor of RNA synthesis in the cells.**

**Measurement of lactate dehydrogenase activity showed these cells to be leaky on incubation at 37°C and thus not suitable for studies of protein secretion.**

SUSPENSIONS of isolated hepatocytes may be used to study the interaction of toxic materials, particularly hepatocarcinogens, with living cells. The cells possess active drug-metabolizing enzymes, such as the cytochrome P450-associated mixed function oxidases (MFO) and the use of hepatocyte suspensions couples the advantages of an *in vitro* system in which multiple experiments may easily be performed, with ability of the cells to metabolize xenobiotics in a manner similar to intact liver. We report here some effects of aflatoxins on isolated hepatocytes prepared by a simplified enzymatic technique.

Investigations using subcellular systems have demonstrated that aflatoxin $B_1$ is metabolized by hydroxylating enzymes in microsomes. One or more of the products can interact covalently with macromolecules such as DNA and protein in mammalian cells and can produce a lethal response in certain strains of bacteria (Garner *et al.*, 1971; Garner, Miller and Miller, 1972; Garner and Wright, 1973; Garner, 1973). Activated products also inhibit RNA and protein synthesis *in vitro* (Moulé and Frayssinet, 1972; Sarasin and Moulé, 1973*b*). We outline here some preliminary investigations on the role of metabolic activation in the inhibition of RNA synthesis by aflatoxin $B_1$ in isolated hepatocytes using an inhibitor of the mixed function oxidases, SKF 525A (diethyl aminoethyl-diphenyl-n-propyl acetate).

### MATERIALS AND METHODS

Heparin used was "Heparin for injection" from Evans Medical Ltd (Speke, Liverpool). Veterinary Nembutal was from Abbot Labs. (Queenborough, Kent). Collagenase was Type I from Sigma (Kingston upon Thames, Surrey) and bovine hyaluronidase was supplied by Miles-Seravac (Holyport, Maidenhead, Berks). Calf serum and Medium 199 were obtained from BDH (Poole, Dorset), Hepes (4-(2-hydroxyethyl)-1-piperazine ethane sulphonic acid) from Flow Labs (Irvine, Ayrshire), and penicillin

* Present address: Chemistry Branch, National Cancer Institute, N.I.H., Bethesda, Maryland 20014, U.S.A.

and streptomycin sulphate from Glaxo (Greenford, Middx).

SKF 525A was a gift from Smith, Kline and French Ltd, Welwyn Garden City, Herts.

Radiochemicals were from The Radiochemical Centre (Amersham, Bucks) and Soluene-100, a commercial sample of solubilizer, was from Packard International (Zurich). The radiochemicals used were (6-$^{14}$C)-orotic acid 60 mCi/mmol, (5-$^{3}$H)-orotic acid 1 Ci/mmol, and $^{14}$C-amino acid mixture (more than 45 mCi/m atom carbon).

Glassware was siliconized with a 2% v/v solution of silicone fluid MS 1107 (Hopkins and Williams, Romford, Essex) in chloroform.

*The preparation of the cell suspensions.*—Animals used were 120 g male C strain albino Wistar rats. Each rat was injected i.p. with heparin and Nembutal (0·05 ml and 0·1 ml/100 g body weight respectively). When the rat was under deep anaesthesia the abdomen was opened wide at right angles to the mid-line, the splenic vein was ligated, and the portal vein was cannulated with a plastic Braunula (size 1 sterile from B. Braun, Melsungen). When the cannula was filled with blood, the rat was transferred to the platform of a specially-designed constant-pressure perfusion apparatus. This apparatus consisted of jacketed upper and lower reservoirs in which perfusate was gassed vigorously with 95% $O_2$/5% $CO_2$ and maintained at 37°C. The initial perfusate was CMFH buffer ($Ca^{++}$- and $Mg^{++}$-free Hanks' buffer prepared from Analar reagents according to Hanks and Wallace (1949) except for the omission of phenol red and chloroform and the $Ca^{++}$- and $Mg^{++}$-salts). Perfusate from the upper reservoir was introduced into the liver through the cannula in the portal vein and the posteria vena cava then severed to allow the perfusate to escape after passage through the liver. The first 50 ml were discarded from an aperture in the base of the platform but after this the perfusate was directed into the lower reservoir from which it was returned to the upper reservoir by a peristaltic pump so that a complete circulation was achieved. At this point a small volume of CMFH containing 50 mg of collagenase and 100 mg of hyaluronidase was added to the lower reservoir. The total circulating perfusate volume was about 100 ml and a constant level was maintained in the upper reservoir 14 cm above the portal cannula, overflow being conveyed directly back to the lower reservoir by a large-bore plastic tube.

Perfusion with the disaggregating enzymes was continued until considerable digestion and disorganization of the liver was evident (10–30 min). The cannula was then removed and the liver rapidly excised into a siliconized 500 ml beaker kept on ice. Perfusion medium was poured on to the liver and gassed with $O_2$/$CO_2$. The knot of connective tissue around the proximal portal branches was grasped with forceps and the liver lobes were gently massaged with a siliconized glass rod. Well-digested livers had a smooth texture and readily produced a creamy suspension with little pressure. The crude cell suspension was transferred to a siliconized flask, gassed thoroughly and shaken for 20 min at 37°C. After incubation, which caused disaggregation of large clumps of cells, the flask contents were again gassed and filtered through nylon bolting cloth (pore size 61 $\mu$m, purchased from Henry Simon Ltd, Cheadle, Cheshire) with the assistance of ice-cold CMFH. The cells were kept on ice for 30 min to allow them to sediment to the base of the flask. The sediment was resuspended in CMFH and collected by centrifugation for 2 min at 30 *g* in siliconized centrifuge tubes. Finally, the cells were again resuspended in CMFH and filtered through bolting cloth, yielding a homogeneous cell suspension.

*Cell viability and yield.*—About 90% of the cells in the final cell suspension had sharply defined peripheries when viewed by phase contrast microscopy and these cells were also viable by the criterion of trypan blue exclusion. Electron microscopy of thin sections of such cells revealed normal ultrastructure, as frequently reported for rat liver cell suspensions prepared by similar means (Berry and Friend, 1969; Capuzzi, Rothman and Margolis, 1971; Müller *et al.*, 1972 and Schreiber, Schreiber and Kartenbeck, 1974).

The yield of viable cells, as determined by haemocytometer counts, was of the order of $2{\cdot}0 \times 10^7$/g wet weight of liver, which represents a recovery of about 20% of the parenchymal cells of the liver (Evans, 1973).

*Incubation of cell suspensions.*—Each incubation contained the following standard components in 100 ml siliconized flasks: 2 ml freshly dissociated cells in CMFH (giving a final viable cell concentration of $2{\cdot}5 \times 10^6$/ml); Full Medium 199, 20 mM with respect to Hepes, pH 7·35; 2 ml calf serum; 0·1 ml standard antibiotic solution (5 mg penicillin, 10 mg streptomycin sulphate, per ml distilled water).

All components and incubation flasks were kept ice-cold until the start of the incubation. Drugs and labelled precursors were added to the incubation mixture in small volumes, usually 0·1 ml or less, from concentrated stock solutions. Incubations were performed at 37°C in a shaking rotary Warburg water bath without gassing.

Time courses of incorporation were followed by removing 1 ml samples and adding these to 1 ml ice-cold 10% w/v trichloroacetic acid. The precipitates were washed by centrifugation ×4 with 5 ml aliquots of ice-cold 5% w/v trichloroacetic acid, dried at 80°C and dissolved in 2 ml Soluene-100. Samples were finally counted in 10 ml of a PPO-based scintillation fluid (which contained, per l, 4 g 2,5-diphenyloxazole (PPO), 100 ml AR methanol, 900 ml AR toluene) in an Intertechnique ABAC SL40. The computing facility of this machine was used to calculate the D/min automatically, after $^{3}$H- and $^{14}$C-quench curves had been determined.

The incorporation of precursors into macromolecules was due to the cells themselves and not to contaminating microorganisms and the incorporation of $^{3}$H-thymidine into DNA during a 2 h incubation was linearly dependent on cell concentration (Evans, 1973).

Serum was found to be necessary to preserve viability during prolonged incubations. In the presence of serum the rate of incorporation of $^{14}$C-orotic acid into RNA during a 40 min time course following a 5·5 h incubation period was 74% of the pre-incubation rate: in the absence of serum the figure was only 28% (Evans, 1973).

*Lactate dehydrogenase* was assayed as recommended by Bergmeyer (1963). The reaction mixture consisted of the following: 2·83 ml phosphate buffer, 0·1 M, pH 7·0; 0·10 ml sodium pyruvate, $9 \times 10^{-3}$ M; 0·05 ml NADH, 10 mg/ml; 0·025 ml test solutions.

The reaction rate was followed by monitoring the decrease in the absorbance at 340 nm with a Unicam SP 8000 spectrophotometer.

*Amidopyrine demethylase*.—The activity of the amidopyrine demethylase in the hepatocytes was estimated by monitoring the quantity of formaldehyde, a product of demethylation, produced on incubation for 2 h with 8·5 mM amidopyrine. Five mM semicarbazide-HCl was included in the incubations to trap the formaldehyde produced. The formaldehyde was assayed using the Nash reagent (0·02 M acetyl-acetone, 0·05 M acetic acid, 2 M ammonium acetate: Nash, 1953) as recommended by Stitzel *et al.* (1966).

Two ml aliquots of the incubates were taken at $t = 0$ and $t = 2$ h and 1 ml of saturated $Ba(OH)_2$ added. The samples were precipitated by the addition of 1 ml of 20% $ZnSO_4$. After centrifugation in a bench centrifuge, 1 ml of Nash reagent was added to 2·5 ml of supernatant. The resulting solution was then incubated at 60°C for 30 min and the absorbance at 412 nm estimated using a Unicam SP 8000 spectrophotometer.

### RESULTS AND DISCUSSION

When the isolated hepatocytes were incubated with low levels of aflatoxin $B_1$, the incorporation of $^{3}$H-orotic acid (an RNA precursor) into acid-insoluble material was very markedly inhibited, whereas the incorporation of $^{14}$C-amino acids (protein precursors) was reduced to a much lesser extent (Fig. 1). Furthermore, there were indications of an effect on the incorporation of orotic acid as early as the time at which the first sample was taken (11 min from the start of the incubations), whereas no effect on the incorporation of amino acids was evident until the elapse of 33–44 min. These observations are consistent with the known effects of aflatoxin $B_1$ on RNA and protein synthesis in rat liver *in vivo*, when an early and profound inhibition of RNA synthesis has been noted (Sporn *et al.*, 1966) prior to an inhibition of protein synthesis (Villa-Trevino and Leaver, 1968).

Inhibition of RNA synthesis by aflatoxin $B_1$ has also been found using Chang liver and kidney T-cells (Scaife, 1971) and kidney epithelial cells (Engelbrecht and Altenkirk, 1972).

FIG. 1.—The effects of aflatoxin $B_1$ on the incorporation of RNA and protein precursors. The contents of the incubations were as in *Methods* with the following additions: ● No additions; □ 0·5% v/v DMF; ▲ DMF + 1·6 × $10^{-6}$ M aflatoxin $B_1$; ○ DMF + 3 × $10^{-6}$ M aflatoxin $B_1$; ■ DMF + 16 × $10^{-6}$ M aflatoxin $B_1$. Each incubation contained 5 μCi of $^3$H-orotic acid and 2 μCi of $^{14}$C-amino acid mixture. All experiments were on the same liver cell preparation.

Similarly, Clifford and Rees (1966) demonstrated that aflatoxin $B_1$, when incubated at a level of 3·2 × $10^{-5}$ M with rat liver slices, caused a 92% inhibition of the incorporation of precursor into RNA after 15 min. Inhibition of incorporation of amino acids into protein was minimal (5%) at this time but increased to 31% after 30 min. As Edwards and Wogan (1970) have pointed out, the inhibition of liver RNA synthesis by aflatoxin $B_1$ is not an artifact of changes in precursor pool sizes or increased RNA breakdown. Likewise, inhibition of protein synthesis in rat liver by aflatoxin $B_1$ cannot be attributed to changes in amino acid uptake or pool sizes (Sarasin and Moulé, 1973*a*).

In order to ascertain further how closely the behaviour of the hepatocyte system reflected the responses of liver cells *in vivo* to treatment with toxins, the effects of four different aflatoxins on the incorporation of RNA precursors was studied. The aflatoxins are known to inhibit the incorporation of RNA precursors into rat liver in the following order of declining potency: $B_1 > G_1 > B_2 > G_2$ (Edwards *et al.*, 1971). In complete agreement with this, the inhibition by $B_1$ was considerably greater than by $G_1$ in the hepatocyte system. $B_2$ and $G_2$ caused hardly any inhibition even though they were present at higher concentrations (Fig. 2).

To determine whether the inhibition of RNA synthesis by aflatoxin $B_1$ requires prior metabolic activation of the carcinogen by the mixed function oxidases of the endoplasmic reticulum, the effects of an inhibitor of mixed function oxidase, SKF 525A (Rogers and Fouts, 1964; Anders and Mannering, 1966; Jenner and Netter, 1972) were investigated. Concentrations of SKF 525A above $1 \cdot 6 \times 10^{-4}$ M completely suppressed all incorporation of $^3$H-orotic acid into acid-insoluble material by the cells (Fig. 3). Cells treated in this way displayed a grossly altered morphology on examination under a light microscope. $1 \cdot 6 \times 10^{-4}$ M SKF 525A caused a 15% inhibition in the rate of incorporation of $^{14}$C-orotic acid. However, in spite of the toxic properties of the inhibitor itself, the pre-incubation of the cells with $1 \cdot 6 \times 10^{-4}$ M or $6 \cdot 6 \times 10^{-5}$ M SKF 525A antagonized to a significant extent the inhibition caused by aflatoxin $B_1$, and this antagonism was greater when the higher concentration of SKF 525A was used. This indicates that at least a part of the inhibition caused by aflatoxin $B_1$ in the cells probably requires the metabolic

FIG. 2.—The effects of different aflatoxins on the incorporation of $^{14}$C-orotic acid into acid-insoluble material. The incubations were as follows: ● No aflatoxin; ▲, $1 \times 10^{-7}$ M aflatoxin $B_1$; ■, $1 \times 10^{-7}$ M aflatoxin $G_1$; ○, $1 \times 10^{-6}$ M aflatoxin $B_2$; △, $1 \times 10^{-7}$ M aflatoxin $G_2$. Each incubation also contained 0·2% v/v methanol (A.R.) and 5 $\mu$Ci of $^{14}$C-orotic acid. All experiments were on the same liver cell preparation.

FIG. 3.—The effect of SKF 525A on the inhibition of incorporation of RNA precursor by aflatoxin $B_1$. The incubations contained: ● No additions; ○ $1{\cdot}6 \times 10^{-4}$ M SKF 525A; □ $1{\cdot}3 \times 10^{-3}$ M and $3{\cdot}3 \times 10^{-3}$ M SKF 525A; ■ $7 \times 10^{-7}$ M aflatoxin $B_1$; ▲ $7 \times 10^{-7}$ M aflatoxin $B_1$ + $1{\cdot}6 \times 10^{-4}$ M SKF 525A; △ $7 \times 10^{-7}$ M aflatoxin $B_1$ + $6{\cdot}6 \times 10^{-5}$ M SKF 525A. Each incubation also included 10 $\mu$Ci of $^3$H-orotic acid and 1% v/v DMF. All experiments were on the same liver cell preparation.

activation of the carcinogen by the MFO-system.

To verify that the SKF 525A was actually inhibiting the drug-metabolizing system, the cells were incubated in the presence and absence of SKF 525A and tested for their ability to demethylate amidopyrine. As shown in Fig. 4, SKF 525A markedly inhibits the demethylation of amidopyrine by the cells, and NADPH greatly stimulates demethylation, showing that low levels of endogenous NADPH may be rate-limiting for the process. Aflatoxin $B_1$, at a level sufficient to cause a significant suppression of RNA synthesis, had no effect upon the demethylation, showing that the drug-metabolizing system is not sensitive to the toxin in the presence of an alternative substrate (amidopyrine).

The observation that SKF 525A could partially relieve the inhibitory effect of aflatoxin $B_1$ on RNA synthesis was confirmed in other experiments, although both the inhibition by aflatoxin and the antagonism of this inhibition by SKF 525A were found to vary from one cell preparation to another. The source of this variation was thought to reside in two uncontrolled factors: (i) differences in the yield of cells from individual livers, and (ii) differences in the integrity of cells in

FIG. 4.—The demethylation of amidopyrine by isolated hepatocytes. Each incubation contained 8·5 mM amidopyrine, 5 mM semicarbazide HCl and 0·2% v/v dimethylsulphoxide in a final volume of 5·5 ml. The incubations also contained: V, $4\cdot6 \times 10^{-7}$ M aflatoxin $B_1$; W, no additions; X, $1\cdot7 \times 10^{-4}$ M SKF 525A; Y, $8\cdot5 \times 10^{-5}$ M SKF 525A; Z, 1 mM NADPH. All experiments were on the same liver cell preparation.

different preparations. Since all the agents added to the system partition between the lipid membrane phase of the cells and the aqueous medium, differences in the total amount of cellular material available in each preparation could influence the local cellular concentration of these materials. Although this factor may be controlled in principle, the packed cell volume of the suspensions used was too small to read accurately using a haematocrit. Damage induced in the cells during preparation, however, cannot easily be controlled, and the influence of a subpopulation of damaged cells on the overall behaviour of the cell suspensions is not known.

For these reasons, comparative experiments using different preparations of cells are not very reliable and are interpretable only if the effects are large. Despite this reservation, the results of one such experiment are presented in Fig. 5. The incorporation of RNA precursor by cells from rats treated *in vivo* with 0·1% phenobarbitone in their drinking water for 1 week was much less sensitive to aflatoxin than incorporation by cells derived from normal rats. *In vivo* studies have shown that phenobarbitone treat-

FIG. 5.—The effects of aflatoxin $B_1$ concentration on the initial rates of incorporation of RNA precursor.

ment protects against the inhibition of RNA synthesis by aflatoxin $B_1$ (Gubmann and Williams, 1970) as well as reducing macromolecular binding of $^{14}C$-labelled aflatoxin $B_1$ (Garner, 1975). The differences involved are large enough to be considered significant. These effects cannot be interpreted with certainty at the present time. They could be due to a change in the metabolite pattern of aflatoxin $B_1$ or alteration of the NADPH/ $NADP^+$ ratio on induction, as suggested by Moldéns *et al.* (1974).

It has been demonstrated that aflatoxin $B_1$ and other carcinogens cause the removal of bound ribosomes from the endoplasmic reticulum of liver cells (Williams and Rabin, 1971; Svoboda and Higginson, 1968). Such a lesion might interfere with the transport of secretory proteins to the exterior of the cell. As a preliminary to testing possible effects on secretion we investigated the integrity of the plasma membranes of the hepatocytes in suspension by measuring the levels of an intracellular enzyme, lactate dehydrogenase (LDH) in both the cellular and extracellular compartments. The results are presented in Fig. 6 and it can be seen that a very appreciable amount of leakage occurs during incubation of these cells.

This experiment demonstrates that the plasma membranes of the hepatocytes isolated by enzyme perfusion are leaky, and this conclusion is supported by other workers who have studied the intactness of the retaining membranes by trypan-blue exclusion (East, Louis and Hoffenberg, 1973). The LDH leakage observed here on incubation of isolated liver cells is very similar to that reported by Johnson, Blecher and Giorio (1972), and would seem to preclude studies of protein export by such cells. The transfer of protein from enzymically-dissociated normal and hepatoma cells to the suspending medium has been investigated by Schreiber *et al.* (1974), but these workers failed to take account of the possible contribution of leakage to their results.

We conclude that the isolated hepatocytes prepared by the simplified enzymatic techniques provided a good system in which to study the effects of toxins and

FIG. 6.—The activity of lactate dehydrogenase in intracellular and extracellular compartments as a function of incubation time at 37 °C. Ten incubations were set up as described in *Methods* and 3 × 2 ml samples taken at timed intervals from particular flasks. These samples were cooled immediately on ice and then centrifuged for 5 min in an MSE bench centrifuge at 4 °C. The pellet was taken as the " cellular " fraction and the supernatant as the " extracellular " fraction. The pellet of cells was made up to 2 ml with incubation medium and sonicated for 30 seconds at full amplitude in an MSE 100W ultrasonic Disintegrator to effect lysis and extrusion of intracellular contents and then recentrifuged. Both supernatant fractions were then frozen prior to an analysis of LDH activity (see *Methods*).

their metabolites on the processes of macromolecular biosynthesis in liver cells. The incorporation of RNA precursor is inhibited by low levels of the hepatotoxin aflatoxin $B_1$ and part of this inhibition can be antagonized by suppressing the activity of the drug metabolizing system. The rate of drug metabolism can be manipulated by inhibition with SKF 525A or stimulation by addition of NADPH. However, the poor integrity of the plasma membranes of the cells precluded meaningful secretion studies.

This work was supported by the Cancer Research Campaign and Medical Research Council and was carried out, in part, in laboratories provided by the Nuffield Foundations. One of the authors (I.H.E.) would like to thank Professor A. P. Mathias and Dr H. Hilderson for advice and encouragement during the development of the cell preparation technique, and the Science Research Council for financial support.

### REFERENCES

ANDERS, M. W. & MANNERING, G. J. (1966) Inhibition of Drug Metabolism. 1. Kinetics of the Inhibition of the N-Demethylation of Ethylmorphine by 2-Diethylaminoethyl 2,2-Diphenylvalerate HCl (SKF 525-A) and Related Compounds. *Mol. Pharmac.*, **2**, 319.

BERGMEYER, H.-U. (1963) Ed. *Methods of Enzymatic Analysis*. New York & London: Academic Press.

BERRY, M. N. & FRIEND, D. S. (1969) High Yield Preparation of Isolated Rat Liver Parenchymal Cells. A Biochemical and Fine Structural Study. *J. Cell Biol.*, **43**, 506.

CAPUZZI, D. M., ROTHMAN, V. & MARGOLIS, S. (1971) Simplified Method for Isolation of Intact Avian and Rat Liver Parenchymal Cells. *Biochem. biophys. Res. Commun.*, **45**, 421.

CLIFFORD, J. L. & REES, K. R. (1966) Aflatoxin: a Site of Action in the Rat Liver Cell. *Nature, Lond.*, **209**, 312.

EAST, A. G., LOUIS, L. N. & HOFFENBERG, R. (1973) Albumin Synthesis by Isolated Rat Liver Cells. *Expl Cell Res.*, **76**, 41.

EDWARDS, G. S. & WOGAN, G. N. (1970) Aflatoxin Inhibition of Template Activity of Rat Liver Chromatin. *Biochim. biophys. Acta*, **224**, 597.

EDWARDS, G. S., WOGAN, G. N., SPORN, M. B. &

PONG, R. S. (1971) Structure-activity Relationships in DNA Binding and Nuclear Effects of Aflatoxin and Analogs. *Cancer Res.*, **31**, 1943.
ENGELBRECHT, J. C. & ALTENKIRK, B. (1972) Comparison of Some Biological Effects of Sterigmatocystin and Aflatoxin Analogues in Primary Cell Cultures. *J. natn. Cancer Inst.*, **48**, 1647.
GARNER, R. C. (1973) Microsome-dependent Binding of Aflatoxin $B_1$ to DNA, RNA, Polyribonucleotides and Protein *in vitro*. *Chemico-Biol. Interactions*, **6**, 125.
GARNER, R. C. (1975) Reduction in Binding of [$^{14}$C] Aflatoxin $B_1$ to Rat Liver Macromolecules by Phenobarbitone Pretreatment. *Biochem. Pharmac.*, **24**, 1553.
GARNER, R. C., MILLER, E. C. & MILLER, J. A. (1972) Liver Microsomal Metabolism of Aflatoxin $B_1$ to a Reactive Derivative Toxic to *Salmonella typhimurium* TA 1530. *Cancer Res.*, **32**, 2058.
GARNER, R. C. & WRIGHT, C. M. (1973) Induction of Mutations in DNA-repair Deficient Bacteria by a Liver Microsomal Metabolite of Aflatoxin $B_1$. *Br. J. Cancer*, **28**, 544.
GUBMANN, M. R. & WILLIAMS, S. N. (1970) Effect of Phenobarbital Pretreatment on the Ability of Aflatoxin $B_1$ to Inhibit Ribonucleic Acid Synthesis in Rat Liver. *Biochem. Pharmac.*, **19**, 2861.
HANKS, J. H. & WALLACE, R. E. (1949) Relation of Oxygen and Temperature in the Preservation of Tissues by Refrigeration. *Proc. Soc. exp. Biol. Med.*, **71**, 196.
JENNER, S. & NETTER, K. J. (1972) Inhibition of Microsomal Drug Metabolism by SKF 525-A. *Biochem. Pharmac.*, **21**, 1921.
JOHNSON, C. B., BLECHER, M. & GIORIO, N. A., JR. (1972) Hormone Receptors. I. Activation of Rat Liver Plasma Membrane Adenylyl Cyclase and Fat Cell Lipolysis by Agarose-glucagon. *Biochem. biophys. Res. Commun.*, **46**, 1035.
MOLDÉNS, P., GRUNDIN, R., VADI, H. & ONENIUS, S. (1974) A Study of Drug Metabolism Linked to Cytochrome P-450 in Isolated Rat Liver Cells. *Eur. J .Biochem.*, **46**, 351.
MOULÉ, Y. & FRAYSSINET, C. (1972) Enzymic Conversion of Aflatoxin $B_1$ to a Derivative Inhibiting *in vitro* Transcription. *FEBS Lett.*, **25**, 52.
MÜLLER, M., SCHREIBER, M., KARTENBECK, J. & SCHREIBER, G. (1972) Preparation of Single-cell Suspension from Normal Liver, Regenerating Liver and Morris Hepatomas 9121 and 5123tc. *Cancer Res.*, **32**, 2568.
NASH, T. (1953) The Colorimetric Estimation of Formaldehyde by Means of the Hautzsch Reaction. *Biochem. J.*, **55**, 416.
ROGERS, L. A. & FOUTS, J. R. (1964) Some of the Interactions of SKF 525-A with Hepatic Microsomes. *J. Pharmac. exp. Ther.*, **146**, 286.
SARASIN, A. & MOULÉ, Y. (1973*a*) *In vivo* Effect of Aflatoxin $B_1$ on Protein Synthesis in Rat Liver. *FEBS Lett.*, **29**, 329.
SARASIN, A. & MOULÉ, Y. (1973*b*) Inhibition of *in vitro* Protein Synthesis by Aflatoxin $B_1$ Derivatives. *FEBS Lett.*, **32**, 347.
SCHREIBER, M., SCHREIBER, G. & KARTENBECK, J. (1974) Protein and Ribonucleic Acid Metabolism in Single-cell Suspensions from Morris Hepatoma 5123tc and from Normal Rat Liver. *Cancer Res.*, **34**, 2143.
SPORN, M. B., DINGMAN, C. W., PHELPS, H. L. & WOGAN, G. N. (1966) Aflatoxin $B_1$: Binding to DNA *in vitro* and Alteration of RNA Metabolism *in vivo*. *Science, N.Y.*, **151**, 1539.
STITZEL, R. E., GREENE, F. E., FUMER, R. & CONAWAY, H. (1966) Factors Affecting the Measurement of Formaldehyde Produced by Enzymatic Demethylation. *Biochem. Pharmac.*, **15**, 1001.
SVOBODA, D. & HIGGINSON, J. (1968) A Comparsion of Ultrastructural Changes in Rat Liver due to Chemical Carcinogens. *Cancer Res.*, **28**, 1703.
VILLA-TREVINO, S. & LEAVER, D. D. (1968) Effects of the Hepatotoxic Agents Retrorsine and Aflatoxin $B_1$ on Hepatic Protein Synthesis in the Rat. *Biochem. J.*, **109**, 87.
WILLIAMS, D. J. & RABIN, B. R. (1971) Disruption by Carcinogens of the Hormone Dependent Association of Membranes with Polysomes. *Nature, Lond.*, **232**, 102.